# Bocavirus Episome in Infected Human Tissue Contains Non-Identical Termini

**DOI:** 10.1371/journal.pone.0021362

**Published:** 2011-06-28

**Authors:** Amit Kapoor, Mady Hornig, Aravind Asokan, Brent Williams, Jose A. Henriquez, W. Ian Lipkin

**Affiliations:** 1 Center for Infection and Immunity, Columbia University, New York, New York, United States of America; 2 Gene Therapy Center, The University of North Carolina at Chapel Hill, Chapel Hill, North Carolina, United States of America; Institut Pasteur, France

## Abstract

Human bocaviruses (HBoV) are highly prevalent human infections whose pathogenic potential remains unknown. Recent identification of the first non-human primate bocavirus [1] in captive gorillas raised the possibility of the persistent nature of bocavirus infection. To characterize bocavirus infection in humans, we tested intestinal biopsies from 22 children with gastrointestinal disease for the presence of HBoV DNA. Four HBoV-positive tissue samples were analyzed to determine whether viral DNA was present in the linear genomic, the episomal closed circular or the host genome-integrated form. Whereas one tissue sample positive for HBoV3 contained the episomal form (HBoV3-E1), none had the genome-integrated form. The complete genome sequence of HBoV3-E1 contains 5319 nucleotides of which 513 represent the non-coding terminal sequence. The secondary structure of HBoV3-E1 termini suggests several conserved and variable features among human and animal bocaviruses. Our observation that HBoV genome exists as head-to-tail monomer in infected tissue either reflects the likely evolution of alternative replication mechanism in primate bocaviruses or a mechanism of viral persistence in their host. Moreover, we identified the HBoV genomic terminal sequences that will be helpful in developing reverse genetic systems for these widely prevalent parvoviruses.

**Significance:**

HBoV have been found in healthy human controls as well as individuals with respiratory or gastrointestinal disease. Our findings suggest that HBoV DNA can exist as episomes in infected human tissues and therefore can likely establish persistent infection in the host. Previous efforts to grow HBoV in cell culture and to develop reverse genetic systems have been unsuccessful. Complete genomic sequence of the HBoV3 episome and its genomic termini will improve our understanding of HBoV replication mechanism and its pathogenesis.

## Introduction

Parvoviruses are small, non-enveloped icosahedral viruses with single-stranded linear DNA genomes that frequently infect animals through the fecal-oral route [2]. They are members of the *Parvoviridae* family, which comprises two sub-families, *Densovirinae* and *Parvovirinae* that infect non-vertebrate and vertebrate hosts, respectively [2], [3]. The International Committee on Taxonomy of Viruses (ICTV) has further classified the sub-family *Parvovirinae* into five genera: Dependovirus, Bocavirus, Erythrovirus, Parvovirus and Amdovirus. Bocaviruses are unique among parvoviruses because they contain a third ORF between the non-structural and structural coding regions [4], [5], [6]. The genus Bocavirus includes the bovine parvoviruses (BPV), minute virus of canines (MVC) [2], porcine bocaviruses [7], gorilla bocavirus [1] and 4 species of human bocaviruses (HBoV 1–4) [4], [8], [9], [10], [11].

Parvoviruses are unique as they have a linear single-stranded DNA genome with hairpin sequences at each end. The length of the DNA genome is between 4500 and 5500 nucleotides [2], [3]. Depending on their replication requirements, parvoviruses (PV) can be autonomous PV or dependoviruses; the latter includes adeno-associated viruses (AAV) and requires presence of a helper virus for successful replication [3]. Bocaviruses are considered autonomous parvoviruses. The non-coding or non-translated regions on the genomic termini contain palindromic sequences, commonly known as inverted terminal repeats (ITR), that play a vital role in viral replication [12]. Since their discovery using nucleic acid amplification-based techniques [4], [8], [9], [11], the terminal regions of all primate bocavirus species remain unsequenced, limiting the scope of studies on viral replication and the development of reverse genetic systems. Here we report the complete genome of the HBoV episome found in infected human intestinal tissue and the secondary structure of its termini.

HBoV1 infection, widely considered acute, has been linked with mild to severe lower respiratory tract infections in children, frequently in association with other viral infections [8], [13], [14], [15], [16], [17], [18], [19], [20]. HBoV1 has also been detected at low frequency in stool samples, although its association with enteric disease is weaker than with respiratory disease [9], [21], [22], [23], [24], [25]. HBoV2 was first identified in stool samples of Pakistani children [11]. Lower frequencies of HBoV2 were detected in the stools of Scottish adults and children [11]. HBoV3 and HBoV2 were recently found in stool samples from Australian children with diarrhea [9], and HBoV4 was first identified in stool samples of children from Nigeria and Tunisia [26]. Whether any of the four HBoV species causes human disease is still undetermined [3], [27], [28].

During a survey of divergent bocaviruses in animals, the first distinct species of non-human primate bocavirus, GBoV1, was identified in stool samples of gorillas from a captive colony [1]. Phylogenetic analysis suggests that GBoV1 is most genetically related to HBoV1. GBoV1 and its related variant were also detected in stool samples of wild animals in Cameroon, confirming their non-human primate origin and worldwide distribution [29]. The presence of GBoV1 in stool samples of animals living in captivity for several years suggests that bocavirus infection may be persistent in these animals. In humans, HBoV infection is predominantly acute; therefore, most studies done to determine the association between HBoV and disease compared the prevalence of viral DNA in samples from symptomatic patients and healthy controls [9], [14], [17], [21], [22], [23], [30], [31], [32], [33], [34]. Here we report the first detection of episomal HBoV3 in human intestinal tissue. These results raise the possibility that bocaviruses, like other well-characterized parvoviruses, may cause persistent infection of their hosts [12], [35].

## Materials and Methods

### Samples and source

Patient biopsies were collected as part of a study to assess the frequency of measles virus transcripts in ilea of children with autistic disorder and gastrointestinal symptoms (AUT-GI) and children with gastrointestinal symptoms without any apparent neurologic disorder (CON-GI) [36]. The Institutional Review Boards (IRB) of Columbia University Medical Center (CUMC) and Partners specifically reviewed and approved the design of the previous study under which these samples were obtained after written consent of parents and guardians, in addition to child assent, as appropriate. After the conclusion of the previous study, all residual samples were de-identified and stored, and thereafter were deemed exempt from additional review by the IRB. All procedures employed in the current study were approved by the CUMC IRB under a human subjects protocol designed for the investigation of properly de-identified samples, wherein the need for any additional consent has been specifically waived. All samples were analyzed anonymously.

### Sample processing and pan-bocavirus PCR assay

DNA was extracted from ileal biopsy material from 22 patients (15 AUT-GI and 7 CON-GI patients) with TRIzol (Invitrogen), using the manufacturer's protocols for sequential extraction of RNA and DNA. DNA concentration and integrity were determined using a Nanodrop ND-1000 Spectrophotometer (Nanodrop Technologies) and a Bioanalyzer (Agilent Technologies). Samples were stored at −80°C. PCR primers panBOV-F1 (5′-TAATGCAYCARGAYTGGGTIGANCC -3′) and panBOV-R1 (5′- GTACAGTCRTAYTCRTTRAARCACCA-3′) were used for the first round of hemi-nested PCR; primers panBOV-F2 (5′- GCAYCARGAYTGGGTIGANCCWGC – 3′) and panBOV-R1 (same as first round) were used for the second round of semi-nested PCR. For the first round of nested PCR, 5 µl of each specimen's DNA were mixed with 5.2 µl 10× polymerase reaction buffer (Qiagen), 3 µl of MgCl_2_, 1.25 µl each dNTP (10 mM), 50 pmol each of forward (both panBOV-F1) and reverse primer (panBOV-R1), 0.65 µl HotStart Taq DNA polymerase (Qiagen) and 32 µl nuclease free water, in a total reaction volume of 50 µl. The PCR reaction was performed using the following protocol: initial denaturation at 95°C for 7 min, followed by 6 cycles at 95°C for 40 sec, 60°C for 45 sec and 68°C for 30 sec, followed by 35 cycles at 95°C for 30 sec, 57°C for 30 sec and 68°C for 30 sec, and a final extension at 72°C for 10 min. During the first round of PCR, the first 6 cycles were done at a high annealing temperature to increase stringency, and the remaining cycles were performed at a lower temperature to facilitate primer hybridization by tolerating some nucleotide mismatches. We added 0.5 µl of the PCR product from the first round to the reaction mixture for the second round of PCR. We used the same cycling conditions for the second round, with an annealing temperature of 64°C for the first 6 cycles and 58°C for the remaining 35 cycles. Following electrophoresis, products were imaged on a 2% agarose gel. PCR products showing positive bands of approximately 290 bp, corresponding to the highly conserved amplified NS gene fragment, were purified using a PCR purification kit (Qiagen) and then directly sequenced from both ends.

### Complete genome sequencing and inverse PCRs

The complete HBoV2 and HBOV3 genomes found in clinical samples were acquired using the primer walking approach as previously described [26]. In brief, each PCR extension step used one primer specific for the novel virus sequence and another degenerate primer to hybridize all known bocavirus sequences. After the complete genome was assembled, each base was sequenced in triplicate to confirm our results.

To detect the presence of the extra-chromosomal circular episomal form of HBoV (HBoV-EPI), we used a nested inverse PCR assay with outward primers against known genomic termini. We used the following inverse PCR primer sequences (5′ to 3′): TATGCTTATAAGTTCCTCTCCAATGGAC for Bo2-Inv-F1; GAAAAGGGTGACTGTAATCCCGAGC for Bo3-Inv-F1; TCTAATTACAGGAGCAGAAAAGGCC for Bo2-Inv-R1; GATTGGCTGACATACGTCACTTCC for Bo3-Inv-R1; GTTCCTCTCCAATGGACAAG for Bo2-Inv-F2; GGGTGACTGTAATCCCGAGCTCA for Bo3-Inv-F2; CAGGAGCAGAAAAGGCCATA for Bo2-Inv-R2; and CTGACATACGTCACTTCCTGGGC for Bo3-Inv-R2. All first round primers were designed to be exonuclease-resistant by phosphorothioation of the first three 5′ end bases. The first round PCR mix contained Bo3-Inv-F1 or Bo2-Inv-F1 and Bo3-Inv-R1 or Bo2-Inv-R1. During the second round, we used either Bo3-Inv-F2 or Bo2-Inv-F2 and Bo3-Inv-R2 or Bo2-Inv-R2. For the first round of nested PCR, 5 µl of DNA extracted from tissue samples were used in first round of PCR using reaction conditions for Hot Start DNA polymerase (as described above). The PCR reaction was performed using the following protocol: initial denaturation at 95°C for 7 min, followed by 5 cycles at 95°C for 40 s, 61°C for 40 s, and 72°C for 1 min, followed by 35 cycles at 95°C for 30 s, 58°C for 40 s, and 72°C for 1.15 min, and a final extension at 72°C for 10 min. We added 0.5 µl of PCR product from the first round to the reaction mixture for the second round of PCR. Second round primers were used under identical cycling conditions, with annealing temperatures of 64°C and 57°C for the first 6 cycles and 60°C and 54°C for the remaining 35 cycles for HBoV3 and HBov2, respectively. Following electrophoresis, products were imaged on a 1.5% agarose gel, isolated, and sequenced from both ends. The complete HBoV3-Episome (HBoV3-E1) genome was submitted to Genbank under accession number JN086998. A detailed protocol of the techniques used to detect the HBoV host chromosome-integrated form (HBoV-INT) can be found in our recent publication on the discovery of mammalian endogenous parvoviruses [37]. Briefly, to detect the genome-integrated form, we used the same nested PCR assays described above, except that the template for the first round of inverse PCR used restriction enzyme digested- and re-circularized host genomic DNA.

### Sequence and DNA secondary structure analysis

To determine the sequence relationship of HBoV3-E1 and HBoV3-IB1 with other known HBoV species, at least one representative virus, including the one with the best-characterized genome, was used to generate the nucleotide sequence alignments of the entire coding region (NS, NP and VP gene). We used the following Genbank sequences in the analysis: EU262978, FJ858259, AB481085, EF450727, AB481077, FJ560720 for HBoV1; FJ170279, EU082213, GQ200737, GU048664, GU048662, EU082214, GU048663, FJ170280, FJ170278 for HBoV2; EU918736, HM132056, FJ948861, GU048665, FJ973562, GQ867667, GQ867666, FJ973563 for HBoV3; and FJ973561 for HBoV4. The model test implemented in the phylogenetic program MEGA 5 [38] showed that the nucleotide substitution pattern among variable sites of different HBoV sequences can be best analyzed using a General Time Reversible (GTR) model with a discrete Gamma distribution (+*G*) of 5 rate categories. We constructed a maximum likelihood phylogenetic tree and then performed bootstrap re-sampling to demonstrate robustness of phylogenetic groupings [38]. Modeling of the secondary structure of the non-coding DNA sequence region (NCR) between the VP and NS genes in HBoV3-E1 and MVC (FJ214110) was done using a standard minimum energy folding algorithm for single-stranded nucleic acids implemented in the “mfold” web server (http://mfold.rna.albany.edu/?q=mfold/DNA-Folding-Form) [39]. For better comparison with the HBoV3-E1 NCR, the secondary structure of the complete MVC NCR was determined after circularizing the linear MVC genome in 5′ to 3′ orientation. Comparative secondary structure analysis of the right-hand termini of different HBoV species was done using HBoV3-E1 and the most complete genomic terminal sequences available in Genbank for HBoV1 (GQ925675) and HBoV2 (GQ200737) [40].

## Results

### Presence of HBoV DNA in human tissue samples

**Figure 1:**
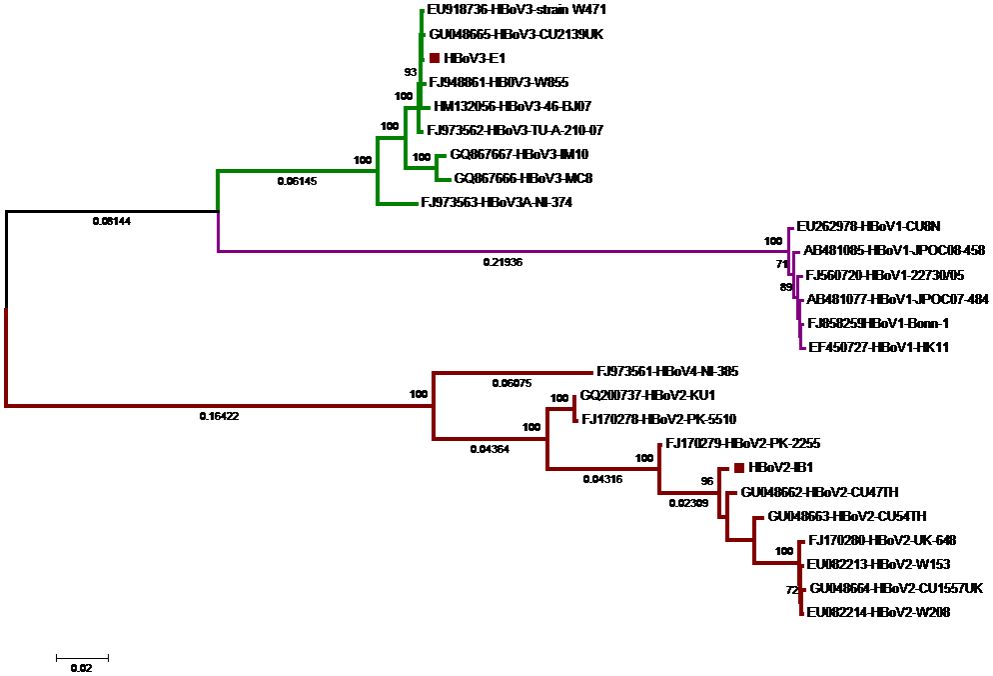
Comparative phylogenetic analysis of HBoV3-E1 and HBoV2-IB1 (filled rectangles). Nucleotide alignments were generated using the complete coding sequence of the NS, NP and VP genes. Names of sequences used for analysis are shown as Genbank accession numbers followed by the names of HBoV species and strains. The tree was constructed with the maximum likelihood method using a GTR+G substitution model [38]. Bootstrap replicates (>70%) are shown above the branches and distances (>0.02) are shown below the braches.

We examined the presence of HBoV DNA in intestinal biopsy samples using a sensitive nested PCR assay (1–10 copies) that targets conserved sequences in HBoV capsid genes and can amplify all known primate bocavirus species [1]. Of the 22 samples tested, 4 tested positive for HBoV DNA. Sequence analysis confirmed that 3 samples were positive for HBoV3, and 1 was positive for HBoV2. These results were confirmed using PCR targeting other regions of the viral genome (NS and NP) [4]. The complete genome of the HBoV2 variant found in one child's biopsy tissues showed >97% nucleotide identity to HBoV2 viruses recently identified in China and Thailand (Genbank accession no. GU048662-3 and GU301644, respectively) (Fig. 1). Similarly, the HBoV3 found in these tissues showed <1–2% nucleotide divergence from published HBoV3 genomes found in Australia, Nigeria and Thailand (Fig. 1). Sequence comparison of HBoV3 viruses found in other children also showed <1% nucleotide divergence over the entire genomic coding region (NS, NP and VP gene). The HBoV sequences reported in this study showed a high sequence similarity to already-published HBoV sequences published by us [4] and others [10], [31], [41]; thus, only minimal phylogenetic analysis was done to confirm the genetic relatedness among these viruses (Fig. 1).

**Figure 2:**
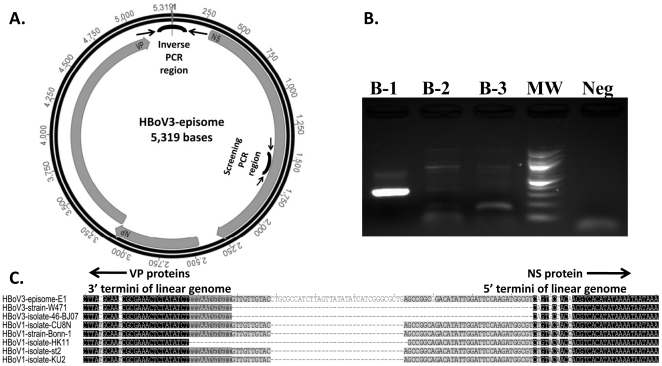
Schematic representation of the HBoV3 episome, results of inverse PCR and sequence alignment of HBoV genomic termini. (A) Describes the genomic organization of the HBoV3 episome and location/orientation of PCR primers used for screening samples and inverse PCR. (B) Shows results of inverse PCR assay for three samples (B-1 to B-3) that were positive for HBoV3 screening PCR with molecular weight (MW) marker and negative (Neg) reagent PCR control. (C) Shows alignment of previously known genomic termini of HBoV3 and HBoV1 linear genomes with the non-coding region of HBoV3 episome. Unique sequence identified in this study is shown as connecting the 3′ and 5′ termini (nucleotide with no background and “-” represent sequence that remained elusive in previous studies).

We used the same nested inverse PCR assays to detect the HBoV-INT and HBoV-EPI forms (Fig. 2A). Non-manipulated nucleic acids extracted from HBoV DNA positive biopsies were used to detect the HBOV-EPI form. Inverse PCR assays of digested and re-circularized nucleic acids were used to detect the HBoV-INT form [37]. Although none of the 4 HBoV DNA-positive samples contained the HBoV-INT form, one sample was positive for the HBoV-EPI form (Fig. 2B). To confirm these results, inverse PCR assays were repeated on the original DNA sample, and the amplification product was sequenced multiple times. The sequences at the ends of the amplification product showed 100% nucleotide identity to the termini of linear HBoV3 and HBoV1 genomes available in Genbank (Fig. 2C). Sequencing and alignment of the inverse PCR product confirmed the amplification of the junction region sequence located between the 5′ and 3′ termini (head-to-tail orientation) and the presence of the HBoV3-EPI form in human tissue (Fig. 2C).

### Genomic characterization of HBoV3 episome

Because we detected the HBoV-EPI form in only 1 sample, we re-extended and re-sequenced the complete HBoV3-E1 genome using the inverse PCR fragment as a starting point for genomic analysis. Every base of the complete HBoV3-E1 genome was sequenced at least 3 times (Genbank accession no. JN086998). The PCR product chromatograms were consistent with infection by a single HBoV lineage. However, mixed HBoV infection cannot be ruled out as population sequencing only allows detection of a minority of variants comprising >10% of the total sequences.

**Figure 3:**
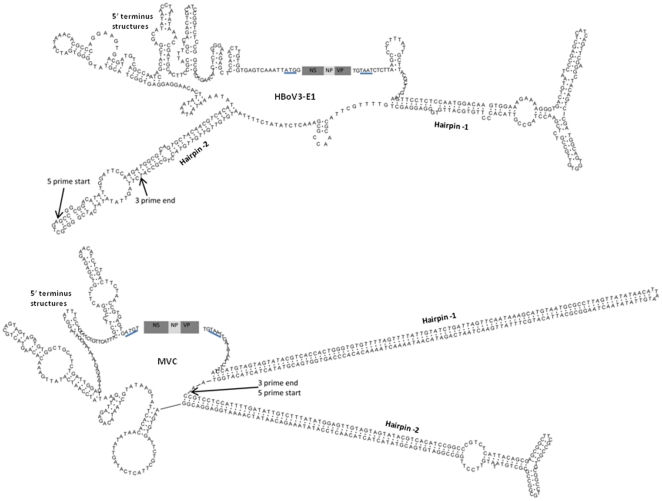
Secondary structure prediction and sequences for the non-coding terminal region of HBoV3-E1 (top) and MVC (bottom). The 3 major bocavirus genes are shown in shaded boxes as NS, NP and VP. The NS gene protein initiation codon and VP genes stop codon are underlined. The 2 stable and long palindromic sequences are shown as hairpin 1 and 2. Cluster of DNA stems and loops immediately upstream of the N-terminus of the NS gene are shown as 5′ terminal structures. Based on the Genbank data, the start and end of the HBoV and MVC linear genomes are shown with arrows.

The complete HBoV3-E1 circular genome contains 5319 nt and codes for 3 large ORFs flanked by a 513 nucleotide long NCR representing both termini (Fig. 2A and 3). ORF1 encodes nonstructural (NS) protein; ORF2 encodes overlapping VP1/VP2 capsid proteins; and ORF3 encodes NP1 [4]. Helicase and ATPase, conserved motifs associated with rolling circle replication, are present within ORF1. Unlike animal bocaviruses, the HBoV3-E1 NS gene also exhibited conserved RNA splicing signals essential to generate shorter and longer NS protein in all HBoV species [1]. To confirm the recombinant origin of HBoV3-E1, we performed a sliding window analysis of sequence homology between the coding region of HBoV3-E1 and members of the other 3 HBoV species. We found that whereas the HBoV3-E1 NS1 and NP1 genes were more closely related to HBoV1 than to HBoV2 and HBoV4, the HBoV3-E1 VP genes were more similar to the HBoV2 and HBoV4 VP1/VP2 gene (data not shown) [4], [9], [10].

As the HBoV3-E1 genome is covalently linked (head-to-tail) and the complete ITR sequences of linear HBoV genomes are not known, we were not able to definitively locate the start or end of the genome (Fig. 3). However, sequence alignment of the complete HBoV-E1 NCR with HBoV linear genomic termini unmasked over 50 previously unsequenced nt in the HBoV termini (Fig. 2C) that is predicted to encode a small 49 aa long protein with very weak domain homology to bacteriophage head-to-tail connecting protein [42]. This family of proteins is found in bacteria and viruses. The head-to-tail connector, a dodecamer of gp10, is embedded within a unique five-fold vertex of the head. The connector serves as the site of assembly of the transient DNA packaging motor that translocates the dsDNA genome to a precursor head shell, or prohead [42]. Additional studies in other HBoV species will be necessary to test the role of this novel protein.

### Sequence and structure of HBoV3-E1 termini

**Figure 4:**
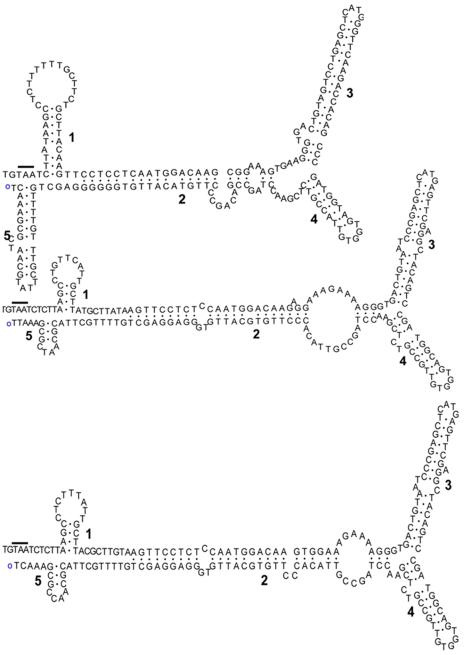
Secondary structure prediction of the sequences immediately downstream of the structural gene of different HBoV species (upper-HBoV1, middle-HBoV2 and lower-HBoV3-E1). The stems and arms are numbered for comparison, and the termination codon of the VP gene is marked by a solid line. The rabbit-ear structure (structure 3 and 4) present in all 3 HBoV species is comparable to similar conserved structures of left-hand side termini reported for animal bocaviruses (MVC and BPV) [44].

Confidence in the predicted secondary structure of DNA or RNA requires comparative modeling of multiple, diverse yet significantly related sequences [43]. As such sequences are unavailable for comparison in HBoV species, we relied on experimentally-confirmed MVC ITR structures [44]. The secondary structure of the 513 nt long HBoV3-E1 NCR sequence between the VP termination and the NS initiation codon contained 3 distinct DNA structures, namely, a hairpin-1, hairpin-2 and a 5′ cluster. Although the MVC and HBoV termini showed no sequence similarity, they shared 3 structurally similar regions in the NCR (Fig. 3). We were unable to locate the terminal nt of the HBoV3-E1 genome. The alignment generated with sequences reported for linear genomes suggests that the terminal bases may be within the hairpin-2 region (Fig. 3). We observed that the hairpin-1 region located in the HBoV3-E1 3′ (RHS) terminus showed remarkable structural similarity to rabbit ear-like structures formed by the termini of several parvoviruses [44]. To confirm the presence of the DNA stem loop, we used the 3′ terminal partial ITR sequence available for all HBoV species for folding and comparative analysis (Fig. 4). Remarkably, despite their sequence diversity, the sequences of all 3 HBoV species folded into almost identical secondary structures. Moreover, HBoV species 1 and 2 are highly genetically diverse and show <80% protein identity, and even lower nt identity, in their protein coding regions (NS, NP and VP gene) [4], [11]. Comparative sequence analysis of the complete 513 nt long HBoV3-E1 NCR showed high sequence identity (>90%) with the partial terminal sequences available for HBoV1, HBoV2 and HBoV3, but not with either of the complete terminal sequences of animal bocaviruses (MVC and BPV). The conserved role of ITR structures in replication of HBoV may require maintenance of a high degree of sequence identity among these genetically diverse viruses. Sequence alignment also confirmed that HBoV termini are non-identical and form imperfect palindromes, characteristics that are more similar to parvoviruses than to animal bocaviruses [12].

## Discussion

GBoV1 is genetically most related to HBoV1 and was first identified in feces of captive animals living in isolation for several years [1]. Later, GBoV1 was also found in fecal samples of wild primates from Cameroon, confirming GBoV1 as an authentic non-human primate bocavirus [1], [29]. These findings inspired us to explore the nature of primate bocavirus infections. Because longitudinal fecal samples from these animals were not available for study, we used human intestinal biopsy samples to characterize the different genomic forms of HBoV.

Our results show that, like adeno-associated viruses [45], HBoV can become persistently established in host cells by forming the extra-chromosomal closed circular (episomal) form. However, unlike AAV, we did not find evidence of HBoV DNA integration into the host genome. The approach we used to determine HBoV integration into human genome is more sensitive than other adapter ligation-based approaches [35], [46], as the primers during 2 rounds of PCR selectively amplify only circular DNA containing viral termini. Previously, we used a similar approach to successfully characterize an endogenous rat parvovirus that exists as a single viral copy per cellular genome [37]. However, we cannot rule out the possibility that HBoV integrates in a rare minority of infected cells. Moreover, because we tested only a few human intestinal tissue samples, a larger sample set should be studied to confirm or refute the existence of the HBoV host chromosome integrated form.

Despite the discovery of different HBoV species, the genomic sequences of their termini remain elusive, limiting the development of a successful cell culture replication system. The information yielded by sequencing the HBoV terminal region and characterizing the HBoV3 episome will be helpful in studying its replication and pathogenesis. The closed circular form of HBoV may be rapidly and clonally amplified using highly efficient strand displacement DNA polymerases to generate large quantities of infective episomes [47]. Unfortunately we didn't have sufficient sample or DNA left to perform these experiments. During replication, most parvoviruses generate replicative intermediates that contain multiple, covalently-linked linear genomes. Notably, these concatamers show head-to-head or tail-to-tail (adjoining of same termini) linear genomic orientations. We sequenced the inverse PCR product multiple times and repeated the assay to confirm the identification of the head-to-tail circularized HBoV genome.

Genomic characterization of HBoV3-E1 resulted in identification of more than 50 previously unsequenced nt in primate bocaviruses [8], [9], [11], [40]. However, it is possible that the linear form of the HBoV genome contains more sequences that maintain terminal stability during replication, that are otherwise not important during replication of a circular template. The AAV episomal junction region lacks some sequences present in linear viral genomes [35]. During the review of our manuscript, a new study [48] reported identification of head-to-tail oriented circular DNA form for another HBoV species (HBoV1) during its infection in cell culture and clinical samples further confirming the existence of HBoV episome in infected cells.

Parvoviruses are ubiquitous and are proposed to contribute to a broad spectrum of diseases in animals, including enteritis, panleukopenia, hepatitis, erythrocyte aplasia, immune complex-mediated vasculitis and cerebellar ataxia [3], [12]. Most studies done to determine the association of HBoV with disease have shown that a high percentage of samples from healthy subjects are also positive for HBoV DNA. However, these studies typically report presence or absence rather than quantitative data. Given our evidence that HBoV sequences may persist, future work of HBoV epidemiology should place more emphasis on the quantity, rather than mere presence, of viral genome. Moreover, previous reports of high prevalence of HBoV in association with infection with other viruses (mixed infections) may reflect reactivation of latent bocavirus episomes.

Although we could not determine what population or percentage of intestinal cells was infected with HBoV in our samples, our results show that, during natural HBoV infection, some linear viral genomes can circularize and exist as extra-chromosomal episomes. Our observation that HBoV genome exist as head-to-tail monomer in infected tissue either reflects the likely evolution of alternative replication mechanism in primate bocaviruses or a mechanism of viral persistence in their host. We also report the complete HBoV episomal genome and the secondary structures of its termini. This novel sequence information will increase our understanding of how these recently discovered parvoviruses replicate and cause infection.
